# Fate of *Clostridium estertheticum* in biofilms and in response to conventional and novel sanitizers

**DOI:** 10.3389/fmicb.2026.1840136

**Published:** 2026-07-22

**Authors:** Weng In Ng, Frances Tran, Hui Wang, Peipei Zhang, M. S. Roopesh, Xianqin Yang

**Affiliations:** 1 Agriculture and Agri-Food Canada, Lacombe, AB, Canada; 2 Department of Agricultural, Food and Nutritional Science, University of Alberta, Edmonton, AB, Canada

**Keywords:** aerotolerant, biofilms, *Clostridium estertheticum*, disinfectants, plasma activated water, spores, UV-C

## Abstract

Blown pack spoilage (BPS) is a major concern to the meat industry and is primarily caused by *Clostridium estertheticum*, a psychrophilic spore-forming, anaerobic bacterium. We investigated the survival mechanisms of *C. estertheticum* and the efficacy of various sanitizers against *C. estertheticum* spores. When cultured alone or with *Pseudomonas poae* at 10°C in a biofilm reactor, viable *C. estertheticum* vegetative cells were recovered only sporadically from stainless steel coupons. However, they were consistently and constantly recovered from planktonic cultures under various conditions for up to 96 h. Spores attached to stainless steel coupons (5.76 ± 0.03 log CFU/cm^2^) were treated with water, PowerFoam, BioDestroy, peroxyacetic acid, UV-C LED, or plasma-activated water. BioDestroy and UV-C reduced (*P* < 0.05) the spore counts by ≥ 4.5 log units, while the other treatments had reductions, if at all, by < 1 log. Heating at 60 or 80°C before UV-C exposure did not affect spore reduction. When *C. estertheticum* spores were co-cultured with *P. poae*, *Leuconostoc gelidum* or *Leu. gelidum* and *Latilactobacillus sakei* in biofilm reactors at 10°C for up to 96 h, recovery of spores from coupons was not affected by incubation time or companion cultures. The companion cultures all developed biofilms. Treatments with BioDestroy or UV-C reduced viable spore counts in biofilms to mostly below the detection level. Interestingly, *P. poae* and *Lat. sakei* may sensitize spores to subsequent treatment. Genome analysis revealed genes encoding biosynthetic gene clusters in these two strains, in particular bacteriocins.

## 1 Introduction

Vacuum packaging is widely applied to fresh meat for shelf life extension, by limiting the growth of strict aerobic microorganisms and selecting for slow growing, obligate and facultative anaerobes that typically have lower spoilage potential (Borch et al., 1996; Li et al., 2022). In fact, chilled, vacuum-packed (VP) meat is the primary form for trading meat within countries and internationally (Pereira et al., 2013). Meat transported overland is usually held at temperatures between 0 and 4°C (James and James, 2002). To further minimize microbial spoilage as well as biochemical processes affecting meat color stability (Jeremiah and Gibson, 2001), a temperature of −1.5 °C, the minimum temperature at which meat can be stored without freezing (Gill et al., 1993), is recommended for long-distance markets. At this temperature, up to 160 days of shelf life has been reported by researchers from Australia (–0.5°C) and Canada (–1.5°C) (Small et al., 2012; Youssef et al., 2014). However, chilled VP meat without temperature abuse ( ≤ 4°C) can be spoiled long before the expected end of shelf life by blown pack spoilage (BPS), a type of spoilage characterized by copious amounts of gas accumulation in the package (Dainty et al., 1989). The time it takes to visible gas accumulation (onset) depends on the number and type of the spoiling organism present, meat pH and storage temperature (Moschonas et al., 2010; Yang et al., 2013). For instance, increase in pack volume by > 50 mL has been reported for VP beef inoculated with *C. estertheticum* at 6 log CFU/cm^2^ and stored at 2°C for 17 days (Yang et al., 2014). With an inoculation level of 3 log CFU/cm^2^, onset to visible gas accumulation has been reported to be 35, 20, 14 days for storage temperatures of −1, 2, and 4 °C, respectively (Moschonas et al., 2010). BPS has been reported worldwide for beef, venison, and lamb (Broda et al., 1996; Byrne et al., 2009; Silva et al., 2011) and, more recently, for pork (Zhang et al., 2020). *Clostridium estertheticum* is the primary BPS causing agent (Cavill et al., 2011) and has the highest capacity to produce gas in VP meat *in vivo*, compared to other psychrotrophic *Clostridium* spp. (Moschonas et al., 2010; Yang et al., 2014). In addition to fresh meat, *C. estertheticum* has also been reported to cause spoilage of sous-vide beef (Dorn-In et al., 2025a).

*C. estertheticum* is an obligate anaerobic, spore-forming bacterium with growth reported at temperatures between −2°C and 22°C (Collins et al., 1992; Yang et al., 2009b). The generation time of the type strain was estimated to be 53.4 h at −2°C and decreased gradually to 11.1 h at 10°C (Yang et al., 2009b). *C. estertheticum* is β-haemolytic (Spring et al., 2003) and able to metabolize lactate, which is abundant in meat, to produce gas (Yang et al., 2009a). This organism has been detected on farms and in abattoirs, from animal derived samples as well as environmental samples, with relatively higher prevalence from fecal materials/animal hides and carcass dressing environments than samples from meat cutting rooms (Angerer et al., 2025; Dorn-In et al., 2025b; Esteves et al., 2021; Moschonas et al., 2009). The primary source for *C. estertheticum* is then likely animal hides, faeces and soil. Even so, Esteves and co-workers examined chilled carcasses and meat products and reported no recovery/detection of the organism on carcasses, but detection on beef products, suggesting recontamination of meat during the fabrication process from environmental sources. This is supported by the detection/recovery of *C. estertheticum* from processing environment surfaces (Angerer et al., 2025; Moschonas et al., 2009).

Bacterial endospores are much harder to kill, compared to their vegetative cells (Setlow, 2014). For *C. estertheticum* spores, Broda has demonstrated that they are resistant to heat, freezing, various chemicals and harsh environments (Broda, 2007). Peroxyacetic acid (PAA), however, reduced *C. estertheticum* spores by 4-log in aqueous suspension (Broda, 2007) at room temperature for 5 min and 2-log on stainless steel coupons at room temperature for 1 min with additional heat shock at 80 °C for 10 min (Mills et al., 2018). Even so, the most commonly used chemical intervention in meat industry to control *C. estertheticum*, fumigation of the facility with PAA during an abattoir’s routine boning room cleaning process, does not always rid the facility of *C. estertheticum*. Ultraviolet light (UV) has long been used for its bactericidal activity, for instance, for decontamination of surfaces, owing to its absence of chemical by-products and compatibility with existing processing line infrastructure (Koutchma, 2008; Koutchma, 2019). Within the entire UV range (100 to 380 nm), the strongest germicidal effect is observed in UV-C (200 to 280 nm). UV-C is widely used to inactivate microorganisms and is produced by mercury lamps. In recent years, UV light-emitting diodes (UV-LEDs) have been replacing mercury lamp based UV-C as a safer alternative. The most effective germicidal wavelength of UV-C LED occurs at a peak of 260 to 265 nm at which DNA absorbs UV the most (Coohill and Sagripanti, 2008). The sensitivity of bacteria to UV-C varies by species; within the spore-forming *Bacillus* spp., bacterial spores are generally 5–10 times more resistant than their corresponding vegetative cells (Coohill and Sagripanti, 2008). In addition to UV-C, plasma-activated water (PAW) has also been investigated for controlling a broad range of bacterial species relevant to food, with efficacy up to 7 log units reduction reported for vegetative bacterial cells (Wang and Salvi, 2021). As with UV-C, available documented accounts on the efficacy of PAW against bacterial endospores are primarily on *Bacillus* spp. Information on effective conventional or novel sanitizers against *C. estertheticum* spores would be of value for the eventual control of BPS. Despite the advances in understanding of gas-forming microorganisms and technology, practical tools to control and prevent remain lacking (Liu et al., 2025).

It has been commonly accepted that *C. estertheticum* survives the aerobic environments in spore form, due to the anaerobic nature of vegetative cells. However aerotolerance of the organism has not been thoroughly examined. In addition, meat fabrication environments are prone to biofilm formation, a response of bacteria to survive a variety of adverse environments (Hall-Stoodley et al., 2004; O’Toole et al., 2000), due to various harsh stressors, influx of bacteria and availability of nutrients and water. The survival and persistence of spoilage as well as pathogenic bacteria in food processing environments has been attributed to biofilm formation (Ban-Cucerzan et al., 2025). Whether *C. estertheticum* can use biofilm as a survival mechanism remains to be investigated. Therefore, the objectives of this study were to investigate the survival mechanism of *C. estertheticum* under aerobic conditions and the efficacy of chemical and novel interventions for inactivation of *C. estertheticum* spores.

## 2 Materials and methods

### 2.1 Bacterial strains and inoculum preparation

Four bacterial strains were used in this study: a strain each of *Clostridium estertheticum* and *Pseudomonas poae*, and two strains of lactic acid bacteria (LAB) of the genera *Latilactobacillus* and *Leuconostoc*. *Clostridium estertheticum* subsp. *estertheticum* (ATCC 51377) was obtained from the American Type Culture Collection (ATCC) and the other three strains were from the culture collection of Lacombe Research and Development Centre (Alberta, Canada). *P. poae* was originally isolated from equipment surface in the fabrication area of a beef packing plant (Wang et al., 2018) and was included as a representative aerobic biofilm former (Visvalingam et al., 2019). *Latilactobacillus sakei* subsp. *carnosus* was originally isolated from VP beef in a previous shelf life study (Zhang et al., 2022). It is the predominant species in the final microbiota on VP beef, outcompeting the inoculated biopreservative *Carnobacterium* spp. The *Leuconostoc* strain was originally isolated from commercial beef primals that had been misted with PAA, vacuum packed and stored for shelf life evaluation (Yang et al., 2021). The genus *Leuconostoc* was predominant on PAA sprayed beef during storage at all chiller temperatures (4°C, 2°C, and −1°C), potentially contributing to shelf life extension. This strain was identified to be *Leuconostoc gelidum* as described in section “2.6 Whole genome sequencing of *Leuconostoc* sp. and *P. poae* and identification of biosynthetic gene clusters.”

For *P. poae*, *Leu. gelidum*, and *Lat. sakei*, stock cultures stored at −80°C were streaked on tryptic soy agar (TSA) and incubated at 25°C for 48 h. A single colony was inoculated to 10 mL of Lennox broth without salt (LB-NS; BD Difco, Fisher Scientific, Mississauga, ON, Canada) and MRS broth, for *P. poae* and the LAB strains, respectively. The cultures were incubated at 25°C with shaking (80 rpm) until stationary phase. The cultures were then diluted in 0.1% peptone water to approximately 10^6^ CFU/mL. Cell density of the cultures was determined by spread-plating aliquots (100 μL) of appropriate dilutions on TSA for *P. poae* and MRS for the two LAB strains. The plates were incubated at 25 °C for 48 h and plates bearing between 25–250 colonies were preferably used for enumeration.

For *C. estertheticum*, 100 μL of a stock culture (vegetative) was directly inoculated into 10 mL of Reinforced Clostridial Medium (RCM; Oxoid, Mississauga, ON, Canada) in Hungate tubes, which was made anoxic by flushing the head space with an anaerobic gas mixture (80% N_2_, 10% CO_2_, 10% H_2_; Air Liquide Canada Inc., Red Deer, AB, Canada). The cultures were incubated at 10°C until stationary phase, and a second transfer in RCM was made, which was incubated at 10°C for 72 h. The resulting cultures were diluted in 0.1% peptone water to approximately 10^5^ CFU/mL and regarded as vegetative cell inoculum in this study. The cell number was determined by spread-plating 100 μL of appropriate dilutions on Columbia agar supplemented with 5% defibrinated sheep blood (CBA; Oxoid). The plates were incubated anaerobically at 10°C for 3 weeks using the BBL GasPak system (Becton Dickinson, Sparks, MD, USA) and enumerated.

*C. estertheticum* spores were prepared as described previously (Yang et al., 2011), with slight modifications. Briefly, a 1 mL-aliquot of *C. estertheticum* inoculum was transferred into serum bottles each containing 75 mL of anaerobic RCM and incubated at 10°C for up to 18 weeks. After 3 weeks of incubation, wet mounts of cultures were periodically examined microscopically under phase contrast illumination. When phase bright spores were seen to be present in > 50% of the cells in a culture, the cells were harvested by centrifugation at 7500 × *g* for 20 min. The pellet was washed four times by resuspending in ice-cold 0.1% peptone water and centrifugation, as before. The spore suspension was heat shocked at 80°C for 10 min to inactivate vegetative cells. Spore counts in the suspensions were determined by spread-plating on CBA as described previously. These spore suspensions were appropriately diluted and used as inoculum for biofilm and sanitizer treatment involving spores.

### 2.2 Survival of C. *estertheticum* vegetative cells under aerobic conditions in monoculture and dual-species cultures with *P. poae*

The ability of *C. estertheticum* vegetative cells to survive aerobic conditions alone or in biofilms of *P. poae* was assessed using a model in-house biofilm reactor (Yang et al., 2023b). The reactor system consisted of a 2 L glass beaker containing food grade stainless steel coupons (grade 301, no. 4 finish; 20 × 25 × 4 mm) held by two circular rings at the bottom. The experiment was carried out under three conditions: (i) *C. estertheticum* alone, inoculated and incubated for 96 h; (ii) simultaneously co-inoculated with *P. poae* and incubated for 96 h; (iii) delayed introduction, where *P. poae* was allowed to form biofilms for 96 h before *C. estertheticum* was introduced. The mixture was further incubated for an additional 96 h. For the *C. estertheticum* alone condition, 10 mL of *C. estertheticum* vegetative cell inoculum at ∼10^5^ CFU/mL prepared as described in section “2.1 Bacterial strains and inoculum preparation” was added to a reactor containing 990 mL LB-NS. For the other two conditions, 10 mL inoculum each for *C. estertheticum* and *P. poae*, and 980 mL LB-NS were used. The reactors were incubated at 10°C under static conditions. At 2 h, 48 h, and 96 h, 1 mL of planktonic cultures and duplicate coupons were withdrawn from each reactor. The original planktonic cultures and appropriate dilutions in 0.1% peptone water were spread-plated on TSA or CBA plates for enumeration of *P. poae* and *C. estertheticum*, respectively. Cells on coupons were detached as previously described (Yang et al., 2023b). Briefly, loosely attached cells were removed by washing each coupon in a 50 mL centrifuge tube containing 15 mL of Phosphate Buffered Saline (PBS) (Becton Dickinson, Sparks, MD, USA) for 1 min. The coupons were then transferred to separate 50 mL centrifuge tubes each containing 1.5 *g* of glass beads and 15 mL of 0.1% peptone water. The attached cells were detached by vortexing at maximum speed for 1 min, and 12 mL of the resulting suspension was collected from each tube and used for enumeration of *P. poae* and *C. estertheticum* by plating.

### 2.3 Efficacy of chemical sanitizers and UV-C LED against *C. estertheticum* spores on stainless steel surfaces

**FIGURE 1:**
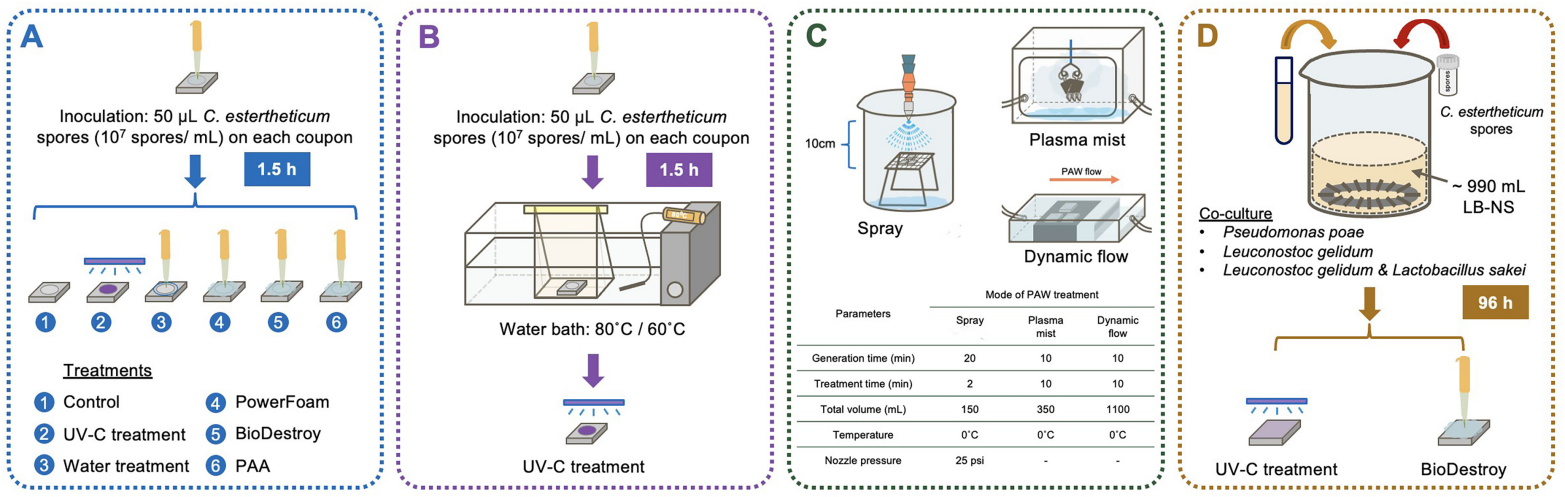
Schematic representation of the experimental frameworks for intervention efficacy against *Clostridium estertheticum* spores. **(A)** Sanitizers and UV-C against spores on stainless-steel surfaces; **(B)** Thermal pre-treatment (80 °C or 60 °C) and subsequent UV-C irradiation against spores on stainless-steel surfaces; **(C)** Set up for three modes of PAW treatment of surface inoculated spores; **(D)** UV-C and BioDestroy against spores embedded within protective multi-species biofilms.

A schematic drawing to demonstrate the experiment set up for sanitizer treatment against spores on stainless steel surface is shown in Figure 1.

#### 2.3.1 Chemical sanitizer treatment

Three chemical sanitizers were tested: PowerFoam plus (Epsilon Chemicals, Canada), a chlorinated alkaline cleaner and degreaser commonly used in meat processing plants; BioDestroy (Sani Marc, Canada), a strong sanitizer formulated to control biofilms; and Peroxy Blast (Epsilon Chemicals, Canada), a PAA-based sanitizer. PowerFoam plus and Peroxy Blast were diluted according to the manufacturers’ instructions on the day of use, in distilled water, to their respective in use concentrations of 2.5% and 200 ppm (0.02%), whereas BioDestroy was directly applied without dilution as per manufacturer’s instructions. BioDestroy is a sanitizer specifically designed for biofilms, containing hydrogen peroxide (10–30%), peroxyacetic acid (5–10%), acetic acid (5–10%), and dodecylbenzenesulphonic acid (10–30%).

An aliquot of 50 μL of spore suspension (10^7^ spores/mL) in 0.1% peptone water was spread evenly onto the top surfaces of stainless steel coupons (10 × 10 × 2 mm). The coupons were air-dried in a biosafety cabinet for 1.5 h with an ambient temperature of 21°C. Sets of three coupons were added with 100 μL of one of the following treatment solutions on the inoculated surface: sterile distilled water, PowerFoam Plus, BioDestroy, and Peroxy Blast. One set of similarly inoculated and air-dried coupons served as control for initial spore counts. After treatment with water or sanitizers for 10 min, coupons were held vertically for approximately 30 s to allow excess liquid to drain. The treated or control coupons were each transferred into a 5 mL centrifuge tube containing 2 mL neutralizing buffer (Difco, BD, Sparks, MD) and 0.2 g sterile glass beads. The tubes were vortexed at maximum speed for 1 min. The resulting suspension was split into two equal proportions, one of which was heat shocked at 80 °C for 10 min and the other remained at room temperature. The reason for the inclusion of heat shock was that in meat processing environment, spores could encounter elevated temperature such as pasteurization of carcasses (Yang, 2017) and heat shrinking of vacuum pack (Moschonas et al., 2011). The suspensions were processed for enumeration of surviving spores by plating.

#### 2.3.2 UV-C LED treatment

The UV-C LED device consisted of a customized LED head (JL3-275S-40; Clearstone Technologies Inc., MN, USA) and a power controller (CF300; Clearstone Technologies Inc.). The LED head had a 5 × 8 array configuration which generated an active area of 2.3 × 3.6 cm^2^ and emitted light pulses at 265 nm. The irradiance of the UV-C LED was determined using an intensity sensor connected to a radiometer (ILT2400; MA, USA). The radiant energy exposure (the energy dosage) of the sample to light at a constant height and exposure time is equal to the product of the irradiance and the exposure time (Almeida et al., 2011). The distance between the LED head and the sample treatment surface was fixed at 4 cm. At this distance, the radiant energy dosages were estimated to be 22.2 J/cm^2^ and 32.0 J/cm^2^ for 10 min of sample exposure to light pulses with power duty cycles of 50% and 80%, respectively. Stainless steel coupons were inoculated with *C. estertheticum* spores as described in section “2.3.1 Chemical sanitizer treatment” and exposed to the LED irradiation for 10 min at 80% duty cycle. The coupons were processed as were for chemical sanitizer treatment for enumeration of surviving spores.

Hot water wash is routinely used in meat processing facility cleaning process, prior to sanitizer application (Yadav et al., 2025). To determine whether prior heat exposure would increase the UV-C susceptibility of spores (Figure 1B), inoculated coupons were individually sealed in sterile bags (7.5 × 12.5 cm; Whirl-Pak) as shown in Figure 1B. The bags were immersed in a water bath at 80°C or 60°C for 10 min or held at room temperature as non-heated controls. The coupons were subsequently exposed to UV-C irradiation for 10 min at 80% or 50% duty cycle and processed for enumeration of surviving spores by plating. No subsequent heat shock at 80°C was applied for post-treatment coupon suspensions.

### 2.4 Efficacy of plasma activated water (PAW) against *C. estertheticum* spores on stainless steel surfaces

Three PAW application modes were evaluated for their efficacy against *C. estertheticum* spores on stainless steel surfaces: spray, mist, and dynamic flow (Figure 1C). PAW was generated on the day of use immediately before the treatment, by cold-plasma bubble discharge in distilled water as described previously (Tiwari et al., 2025). Sterile distilled water applied under identical conditions served as the corresponding control.

For spray treatment, a set of three inoculated coupons were placed on a wire-gauze stand inside a 4 L glass beaker. Coupons were sprayed for 1 min with sterile distilled water as shown in Figure 1C, Followed by 1 min with PAW using a medium-flow spray nozzle operated at 25 psi as measured by a pressure regulator and positioned approximately 10 cm from the coupon surface. For mist, a set of three coupons were clipped onto a holder and suspended 8 cm below the top of a sealed mist chamber. PAW was introduced through two opposing inlet ports for 10 min, producing a uniform mist that enveloped the coupons. For dynamic flow, a set of three coupons were placed on a flat plastic holder within a customized flow-through system. For dynamic treatment, PAW (1100 mL) was circulated continuously for 10 min, flowing from an upper reservoir across the coupon surfaces and into a lower collection chamber to simulate a dynamic wash. PAW treatment was carried out at the University of Alberta. Following each PAW or water control treatment, coupons were transferred into 5-mL tubes containing 2 mL neutralizing buffer and sterile glass beads, placed on ice and transported to the Lacombe laboratory. The samples were processed to enumerate the surviving spores within 3 h of treatment.

### 2.5 Fate of *C. estertheticum* spores in multispecies biofilms and their response to UV-C and BioDestroy treatment

The fate of *C. estertheticum* spores under co-culture conditions was assessed with (i) *P. poae*, (ii) *Leu. gelidum*, or (iii) *Leu. gelidum* and *Lat. sakei* using the in-house biofilm reactors. For each biofilm reactor, 10 mL inoculum each for the three companion cultures was used. *C. estertheticum* spores (∼1 × 10^8^ spores/mL) were added at 1 mL per 1L reactor containing LB-NS (990 mL) as shown in Figure 1D, yielding an initial concentration of ∼10^5^ spores/mL. Biofilms were allowed to develop for 96 h and planktonic cultures and coupons were sampled as at 2 h, 48 h and 96 h as described in section “2.2 Survival of C. *estertheticum* vegetative cells under aerobic conditions in monoculture and dual-species cultures with *P. poae*.”

Efficacy of UV-C and BioDestroy against spores in 96 h-biofilms was assessed (Figure 1D). Each coupon was gently rinsed in 15 mL of PBS for 1 min to remove loosely associated cells. After dripping off excess solution, the coupons were subjected to BioDestroy or UV-C LED irradiation at an 80% duty cycle as described in section “2.3 Efficacy of chemical sanitizers and UV-C LED against *C. estertheticum* spores on stainless steel surfaces”. Untreated coupons served as control. Surviving spores were detached and enumerated by plating as before.

### 2.6 Whole genome sequencing of *Leuconostoc* sp. and *P. poae* and identification of biosynthetic gene clusters

To isolate the predominant *Leuconostoc* spp. from VP primals in the previous shelf life evaluation study (Yang et al., 2021), an aliquot of the purge samples from each of five packages (one from 4 °C and two each from 2 °C and −1 °C) were subjected to lactic acid bacteria isolation and purification using de Man, Rogosa, Sharpe (MRS; Thermo Scientific Oxoid, Mississauga, ON, Canada) agar. Three isolates from each package were selected for DNA extraction, using a QIAGEN DNeasy Blood & Tissue kit (QIAGEN, Toronto, ON, Canada) following the manufacturer’s instructions for Gram-positive bacteria. A shotgun library was constructed and sequenced using an in-house Illumina MiSeq PE250 bp platform. The *P. poae* strain was included in a different batch of sequencing using an Illumina HiSeqX platform by Genome Quebec (Montreal, Quebec, Canada). The quality control and trimming of the raw sequencing reads, and subsequent genome assembly was carried out as described previously (Yang et al., 2023a). The species identity of each isolate was determined using the toolkit GTDB-Tk which uses the Genome Database Taxonomy (GTDB) (Chaumeil et al., 2019). Sequencing and genome assembly for *Lat*. *sakei* were reported previously (Zhang et al., 2022). To determine whether the three companion bacterial strains harbor genes encoding for bioactive compounds, the genome assemblies were subjective to antiSMASH (Blin et al., 2023) to detect and characterize biosynthetic gene clusters (BGCs).

### 2.7 Statistical analysis

All bacterial counts were log_10_-transformed prior to analysis. Samples with no detectable colonies on agar plates were assigned values 0.5 log units below the detection limit. Counts were expressed as log_10_ CFU/mL for planktonic cultures and log_10_ CFU/cm^2^ for coupon samples. Two-way ANOVA was performed in R (v4.4.3; (R Core Team, 2021)) to evaluate the effects of treatment and time, followed by Sidak *post hoc* tests for pairwise comparisons. Differences were considered statistically significant with *P* < 0.05.

## 3 Results

### 3.1 Survival of *C. estertheticum* vegetative cells under aerobic culture conditions

The survival of *C. estertheticum* vegetative cells in planktonic cultures and on stainless steel coupons was assessed for up to 96 h in biofilm reactors under three inoculation regimes (*C. estertheticum* alone, simultaneous co-inoculation with *P. poae*, and delayed introduction of *C. estertheticum* to 4-day old *P. poae* cultures). Viable cell counts in the inoculum were 5.31 ± 0.56 log CFU/mL for *C. estertheticum* and 6.28 ± 0.07 log CFU/mL for *P. poae*, corresponding to initial counts of 3.3 and 4.3 log CFU/mL in the biofilm reactor. Despite *P. poae* being classified as a strict aerobe, it could grow on CBA plates incubated anaerobically (Supplementary Figure 1). However, the colonies of *C. estertheticum* and *P. poae* on CBA plates were readily distinguishable. For all three conditions, *C. estertheticum* displayed limited attachment to stainless steel surface. Of the coupons from all conditions and incubation times, only 19.4% yielded *C. estertheticum* counts and they were ≤ 4 CFU for the undiluted coupon suspension. For the planktonic cultures, however, *C. estertheticum* counts ranged from 2.66 ± 0.47 CFU/mL to 3.81 ± 0.21 CFU/mL without significant differences among culture conditions or time points (*P* > 0.05). For *P. poae*, the viable cell counts recovered from coupons under simultaneous co-inoculation were 3.04 ± 1.07, 4.94 ± 0.57, 5.63 ± 0.45 CFU/cm^2^ for 2 h, 48 h and 96 h, respectively. For delayed introduction of *C. estertheticum*, the counts for *P. poae* were 2.56 ± 0.50, 5.99 ± 0.09, 6.09 ± 0.23, and 6.62 ± 0.21 log CFU/cm^2^ for 2 h, 96 h+2 h, 96 h+48 h, and 96 h+96 h, respectively. The planktonic cultures had initial *P. poae* counts of 4.3 log CFU/mL at 2 h, and between 7.5 to 8.5 log CFU/mL for all other time points.

### 3.2 Efficacy of chemical sanitizers, UV-C-LED, and PAW against surface attached *C. estertheticum* spores

**FIGURE 2:**
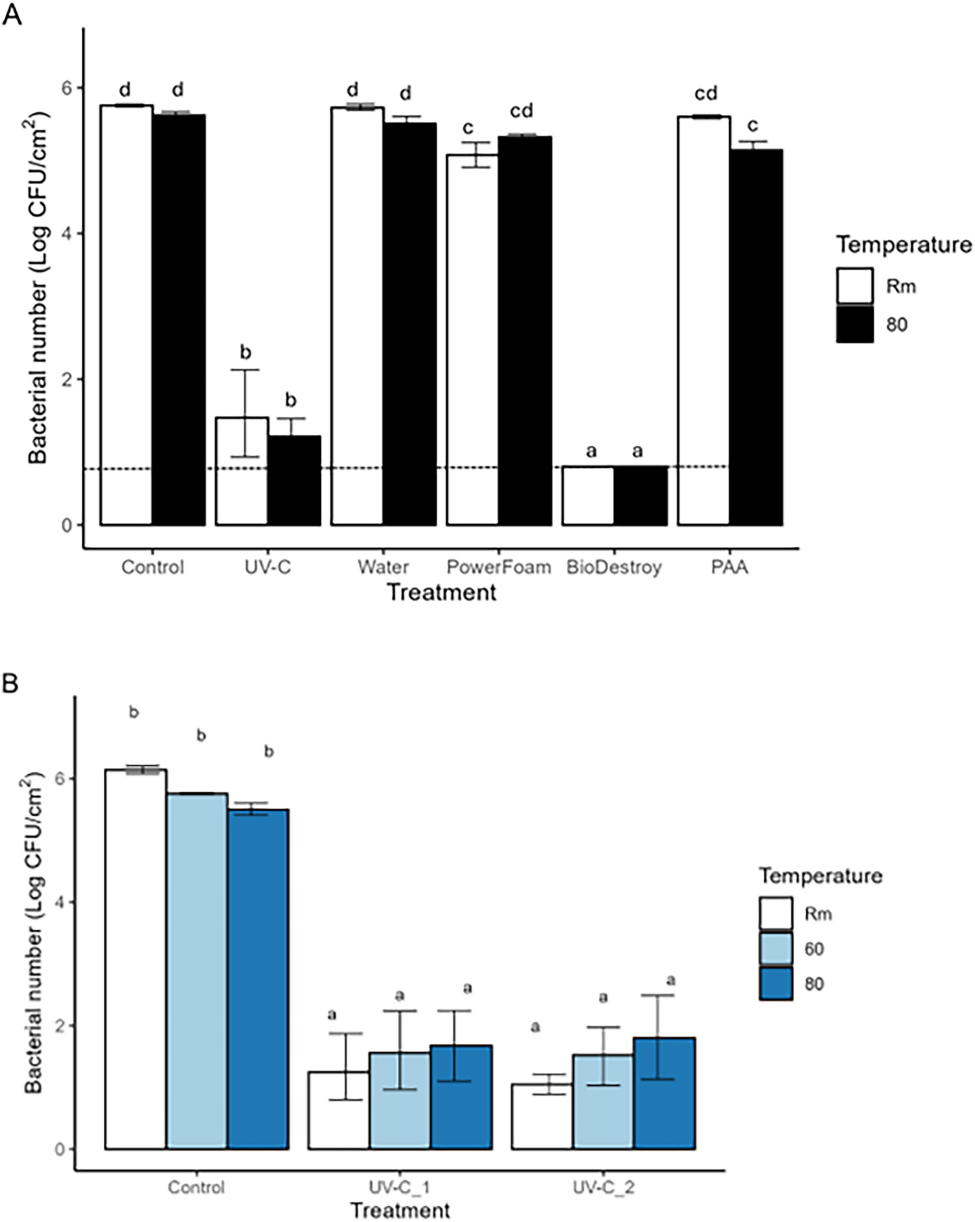
Inactivation of *C. estertheticum* spores on stainless steel coupons by UV-C LED and chemical treatments **(A)** and by heating and subsequent UV-C LED **(B)**. **(A)** surface attached spores were not or were treated with UV-C, Water, PowerFoam, BioDestroy or a peroxyacetic acid (PAA) based sanitizer. Spores detached from coupons were left at room temperature (Rm) or incubated at 80 °C for 10 min (80). **(B)** Surface attached spores were left at room temperature (Rm) or incubated at 60 °C or 80 °C prior to UV-C LED exposure at two dosages: 22.2 J/cm^2^ (UV-C_1) and 32.0 J/cm^2^ (UV-C_2). Columns in each panel under the same temperature that do not share the same letter were significantly different (*p* < 0.05).

Figure 2A summarizes the survival of surface attached *C. estertheticum* spores after UV-C LED and chemical treatments. The spore count on untreated coupons was 5.76 ± 0.03 log CFU/cm^2^. UV-C and Powerfoam reduced (*P* < 0.05) viable spore counts by 4.5 and 0.7 log units, respectively; while none of the samples from BioDestroy treated coupons yielded viable spore counts, indicating a reduction of > 4.6 log. For the UV-C LED treated coupons, only 50% yielded counts. PAA or water, on the other hand, did not result in any significant reduction in spore counts. Interestingly, a further heating at 80°C for 10 min resulted in a small ( < 0.5 log) but significant (*P* < 0.05) decrease in viable spore counts for the control, water, and PAA treatments, but not for UV-C LED or PowerFoam. Even so, the difference may not be practically meaningful due to the limitations of the plating method.

The effect of sequential treatment of heat and UV-C LED on surface attached *C. estertheticum* spores is shown in Figure 2B. Viable spore counts were reduced by 5.09, 4.62 and 4.34 log by UV-C LED only, heat shock at 60°C and UV-C LED, and at 80°C and UV-C LED, respectively, with the radiant energy dosage from UV-C LED exposure estimated at 32.0 J/cm^2^. UV-C LED exposure reduced spores on coupons after heat treatment at 60°C and 80°C by 4.24 and 3.70 log units. A lower UV-C LED exposure dose of 22.2 J/cm^2^ was similarly effective in inactivating *C. estertheticum* spores as the higher dose of 32.0 J/cm^2^ (Figure 2B).

For PAW treatment under spray mode, the mean log counts were 3.07 ± 0.55 and 4.08 ± 0.08 log CFU/cm^2^ for treatment and water control. The corresponding numbers for the plasma mist and dynamic flow mode were 5.27 ± 0.38 and 5.75 ± 0.35 log CFU/cm^2^, and 5.02 ± 0.01 and 5.27 ± 0.02 log CFU/cm^2^. The untreated coupons had a mean count 5.68 ± 0.08 log CFU/cm^2^.

### 3.3 Fate of *C. estertheticum* spores in mixed species biofilms

**FIGURE 3:**
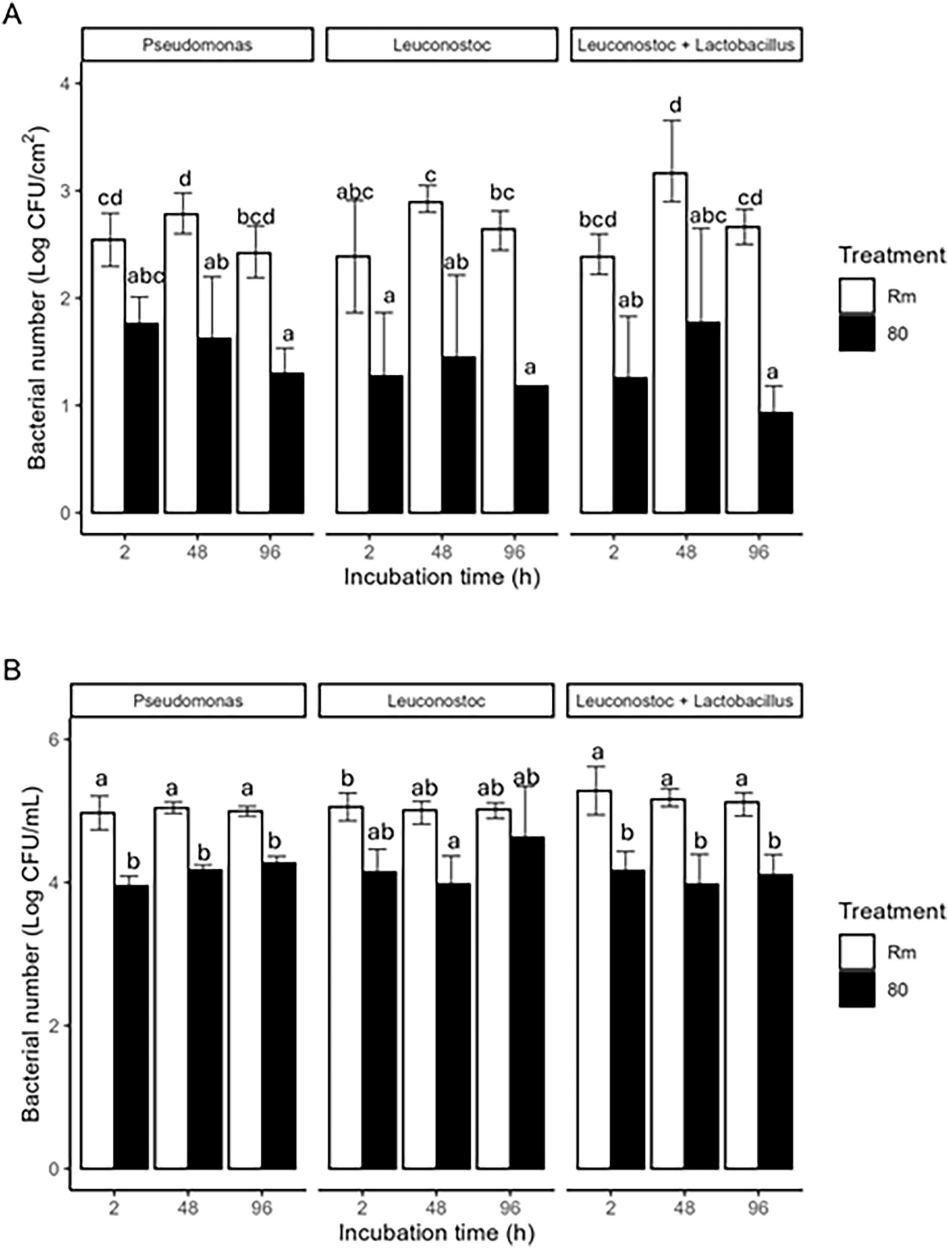
Survival of *C. estertheticum* spores in biofilms **(A)** and planktonic cultures **(B)** when co-cultured with *Pseudomonas*, *Leuconostoc*, and *Leuconostoc* + *Lactobacillus*. Samples were not (Rm) or were subjected to heating at 80 °C prior to plating (80). Different lowercase letters denote significant differences among treatments and time points within each panel (*p* < 0.05).

Colonies of *Leu. gelidum*, *Lat. sakei* and *C. estertheticum* were readily distinguishable on TSA and CBA plates (Supplementary Figure 2). Overall, the companion culture or incubation time did not affect the number of viable spores recovered from biofilms on stainless steel coupons, with the numbers ranging between 2.5 log CFU and 3.2 log CFU/cm^2^ (Figure 3A). When the spore suspension from coupons was subsequently heated at 80°C 10 min, the viable spore counts for 48 h and 96 h were significantly lower (*P* < 0.05) for all three co-cultures than their corresponding unheated counterparts, by 1.1–1.7 log units. A similar trend was observed for the viable spore counts in planktonic cultures with *P. poae* and *Leu. gelidum* + *Lat. sakei* except that the subsequent heat treatment also reduced (*P* < 0.05) spore counts at 2 h (Figure 3B).

For the companion cultures, *P. poae* increased from ∼3.1 log CFU/cm^2^ to ∼5.6 log CFU/cm^2^ on stainless coupons and 4.61 ± 0.13 to 8.11 ± 0.09 log CFU/mL in planktonic cultures. The corresponding numbers were 1.93 ± 0.38 to 5.91 ± 0.21 log CFU/cm^2^, and 4.62 ± 0.59 to 8.57 ± 0.19 log CFU/mL for *Leu. gelidum*. In the tri-species cultures, the numbers for *Leu. gelidum* and *Lat. sakei* were largely similar to those for *Leu. gelidum* in dual-species cultures. Heating at 80°C eliminated all three companion cultures either in planktonic cultures or in biofilms.

### 3.4 UV-C vs BioDestroy against *C. estertheticum* spores in multi-species biofilms

**FIGURE 4:**
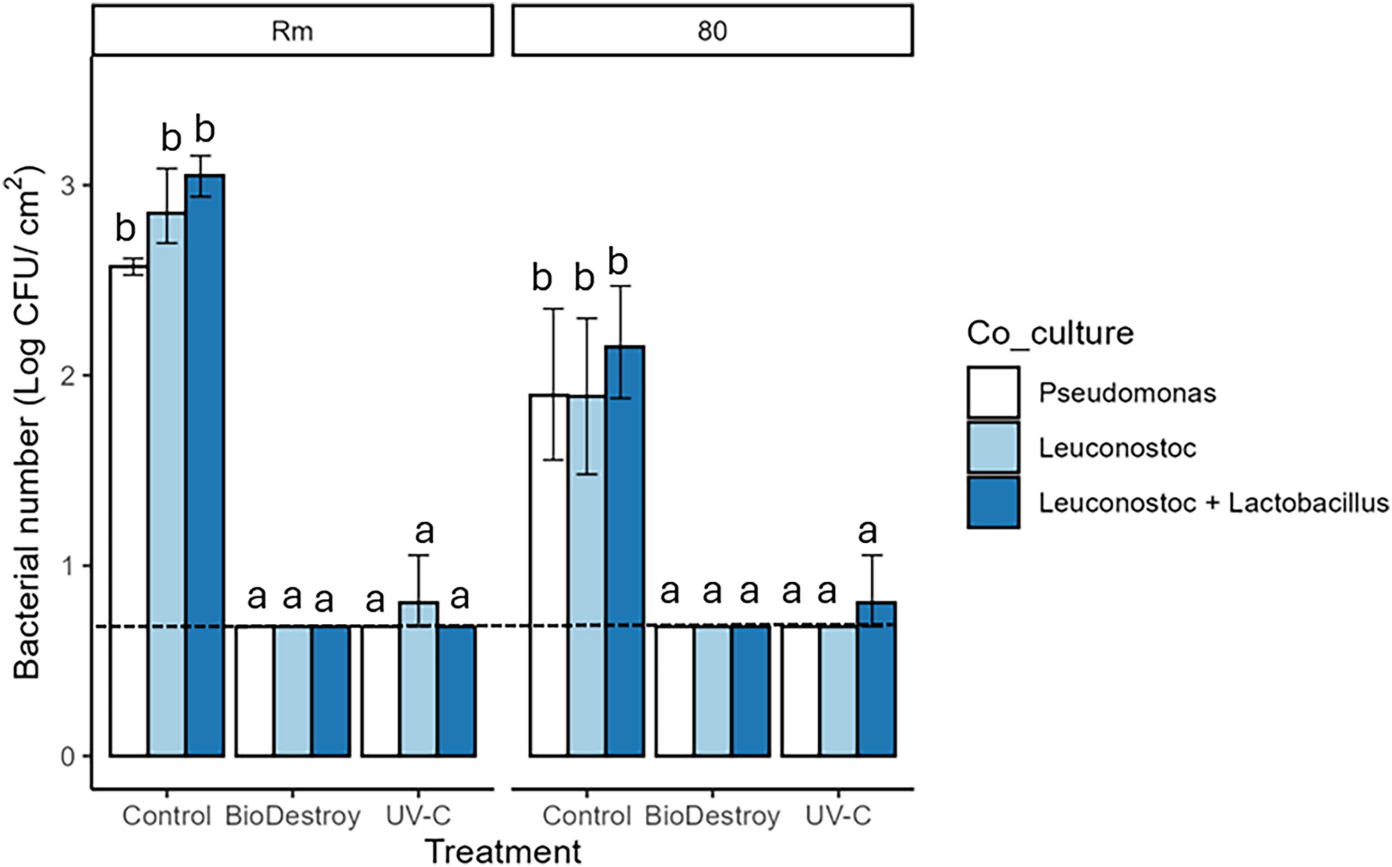
Efficacy of UV-C LED and BioDestroy against *C. estertheticum* spores in biofilms of *Pseudomonas*, *Leuconostoc*, or mixed *Leuconostoc* + *Lactobacillus*. Samples were not (Rm) or were subjected to heating at 80 °C prior to plating (80). The dash line indicates the detection limit of 1.18 log CFU/cm^2^. Different lowercase letters denote significant differences among treatments within each panel (*p* < 0.05).

Figure 4 shows the efficacy of UV-C LED or BioDestroy treatments against *C. estertheticum* spores in 96-h biofilms formed with *P. poae*, *Leu. gelidum*, or *Leu. gelidum* + *Lat. sakei*. Both UV-C LED and BioDestroy eliminated *C. estertheticum* spores in all mixed species biofilms except one sample where 1 CFU was recovered from one UV-C LED treated coupon on CBA plate after incubation at 10°C for 3 weeks. The combination of UV-C or BioDestroy and subsequent heat shock at 80°C reduced viable spore counts to a non-detectable level for all except one coupon from tri-species biofilms where 1 CFU was recovered.

### 3.5 Biosynthetic gene clusters (BGC) in the three companion strains

**FIGURE 5:**
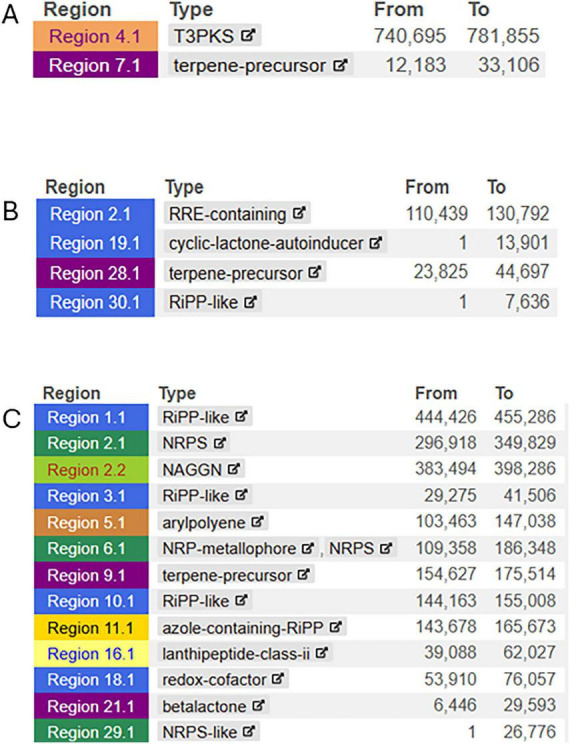
Secondary metabolite biosynthetic gene cluster (BGC) in *Leuconostoc gelidum* **(A)**, *Latilacotobacillus sakei* **(B)**, and *Pseudomonas poae* **(C)** genomes as predicted by antiSMASH.

The 15 *Leuconostoc* isolates were clonal with average nucleotide identity (ANI) > 99.99% and belong to *Leuconostoc gelidum*. The *Leu. gelidum* genome contained genes encoding for type III polyketide synthases (T3PKS) and a terpene precursor (Figure 5A). In addition to genes encoding class II bacteriocins (ribosomally synthesized and post-translationally modified peptides, RiPPs) reported previously (Zhang et al., 2022), the *Lat. sakei* genome also harbors BGCs encoding cyclic-lactone autoinducer and terpene precursor (Figure 5B). For *P. poae*, 13 BGCs were detected (Figure 5C), encoding for bacteriocins (RiPPs and lanthipeptide-class II; *n* = 5), beta-lactone (*n* = 1), non-ribosomal peptide synthetase (NRPS; *n* = 3), N-acetylglutaminylglutamne amide (NAGGN; *n* = 1), arylpolyene (*n* = 1), terpene precursor (*n* = 1) and redox co-factor (*n* = 1).

## 4 Discussion

*C. estertheticum* is the major culprit for BPS, a type of premature spoilage of chilled VP meat from the major livestock species beef, pork, lamb, and venison worldwide (Broda et al., 1996; Byrne et al., 2009; Silva et al., 2011; Zhang et al., 2020). Since the first report in 1989 (Dainty et al., 1989), much of the research has focused on the characteristics and causative agents of BPS. Effective and practical control measures are still lacking. In the present work, we investigated the mechanisms of survival of *C. estertheticum* alone or with companion bacteria, and the efficacy of physical and chemical disinfectants.

*Clostridium* is a genus of spore-forming, obligate anaerobes, with members adapted to diverse habitats (Cruz-Morales et al., 2019). By virtue of being in the *Clostridium* genus, it has been commonly believed that *C. estertheticum* survives the natural environment primarily in its spore form and consequently spores must be present on meat at the time of packaging, in order to cause BPS. In the present work, approximately 50% of the vegetative cells of *C. estertheticum* were recoverable on CBA plates after incubation in LB-NS at 10°C for up to 96 h with or without the companion culture of *P. poae*. In fact, vegetative cells of *C. estertheticum* as well as those of 10 other psychrotolerant clostridia species proliferated on VP meat of various pH, with *C. estertheticum* leading to copious gas production at meat pH between 5.5 and 5.9 (Yang et al., 2014). Vacuum pouches made of an ethylene-vinyl alcohol copolymer/nylon laminate are commonly used for meat, and such pouches do not completely block oxygen transmission (Gill and Gill, 2009). Despite the remaining respiratory activity of meat, VP meat would unlikely be an oxygen free environment. As such, aerotolerance of *C. estertheticum* vegetative cells should be regarded as a survival mechanism for the anaerobic organism *C. estertheticum* in the natural environment. Other members of the *Clostridium* genus such as *C. sporogenes* and *C. perfringens* have also been reported to be aerotolerant (Briolat and Reysset, 2002; Sadr et al., 2024).

Bacterial endospores are the most resistant living structures known, owing largely to their multilayered structure, particularly the thick spore coat and cortex (Atrih and Foster, 2002). Of the biocides explored in the present study, BioDestroy and UV-C LED were highly effective in inactivating spores attached to stainless steel surfaces ( > 4 log unit reduction) or in multispecies biofilms (mostly to below the detection limit). It has been suggested that the mode of action of oxidizing agents against bacterial endospores is to cause damage to the spore’s inner membrane, a membrane whose integrity is essential for spore viability, and to sensitize spores to subsequent stress (Cortezzo et al., 2004). The effectiveness of BioDestroy is likely due to its high concentrations of the oxidizing agents hydrogen peroxide and peroxyacetic acid, and the additional acidic stressor acetic acid and the surfactant dodecylbenzenesulphonic acid. Broda (2007) reported that incubation of *C. estertheticum* spores suspended in PAA at 180 ppm at room temperature for 5 min resulted in a > 4 log reduction. Mills and co-workers found spraying PAA at 180 ppm for 1 min and a subsequent heat shock at 80°C for 10 min reduced spore counts on stainless steel by 3 log (Mills et al., 2018). In the present study, incubation of *C. estertheticum* spores on stainless steel surfaces with 200 ppm PAA for 10 min was largely ineffective. The differences between the published accounts and the present work in the efficacy of PAA against *C. estertheticum* spores may be attributable to differences in experimental set up. Surface attached organisms are generally found to be less susceptible to disinfectants than suspended organisms under the same concentrations (Gibson et al., 1995). Even so, the further reduction by heating PAA treated spores at 80°C for 10 min (Figure 2A) is in agreement with the proposed sensitization of spores by oxidizing agents to subsequent stressors (Cortezzo et al., 2004). For chlorine solutions, HOCl is the predominant form in the solution at low pH (Gill et al., 1993; James and James, 2002; Jeremiah and Gibson, 2001) and OCL^–^ at higher pH. PowerFoam contains potassium hydroxide and sodium hypochlorite, which has been demonstrated to be effective in reducing bacterial numbers on conveyor belts in commercial beef processing facilities (Yadav et al., 2025), likely through the emulsifying effects of OCL^–^, rather than the oxidizing effect of HOCl. The proteinaceous nature of the spore coat may be less susceptible to the emulsifying effect, contributing to the limited effect of PowerFoam on *C. estertheticum* spores. PAW contains multiple reactive species, such as hydrogen radical (∙OH), hydrogen peroxide (H_2_O_2_), and ozone (O_3_), and reactive nitrogen species such as nitrites (NO_2_^–^), nitrate (NO_3_^–^); their respective concentrations are often below 1 ppm (Tiwari et al., 2025). The spray mode of PAW resulted in a 1 log reduction compared to its water control. Future studies may focus on longer PAW generation times, which could lead to higher ROS levels and enhance the activation of *C. estertheticum* spores.

In the present study, exposure to UV-C LED at a dosage at 22.2 J/cm^2^ reduced *C. estertheticum* spores attached to stainless steel surfaces by > 4 log units and in multispecies biofilms to mostly below the detection limit. For clostridial spores, one study demonstrated that UV-C radiation was effective in eliminating *C. difficile* from touch surfaces under laboratory conditions simulating hospital settings (Różańska et al., 2025), while another study suggested it was largely ineffective following routine daily cleaning with sodium hypochlorite at a hospital (Fourman et al., 2026). Gayán et al. (2013) investigated UV-C inactivation of spores of various *Bacillus* spp. and reported reductions between 2.3 and 4.1 log at 23.72 J/mL. A lack of sensitization to UV-C by prior heat treatment has also been reported by those researchers, which is consistent with the present study as presented in Figure 2B. It is plausible that the prior heat shock did not lead to structural change to allow more effective treatment by the subsequent UV-C exposure. On the other hand, Gayán et al. found sensitization of spores to heat after UV-C treatment (Gayán et al., 2013). The findings of the present study and those in previous reports show that UV-C can be effective in reducing bacterial endospores and may be explored as a sanitization strategy for equipment surfaces in meat processing facilities.

In the natural environments such as meat processing facilities, *C. estertheticum* likely co-exist with other background microbiota as diverse taxa have been found on various surfaces in the facilities before and after sanitization (Wang et al., 2018; Yadav et al., 2025). In the present study, *C. estertheticum* spores integrated into biofilms of *P. poae*, *Leu. gelidum*, and *Leu. gelidum* and *Lat. sakei*. It is interesting that the co-cultures in biofilms and in planktonic form sensitized the spores to subsequent heat treatment, more consistently for *P. poae* and *Leu. gelidum* and *Lat. sakei* than with *Leu. gelidum* alone. The exact mechanism of such sensitization is unclear. Bacteriocins produced by various microorganisms have shown sensitization of spores of *Bacillus* spp. and *Clostridium* spp. (Egan et al., 2016). The sensitization by *Lat. sakei* and *P. poae* of *C. estertheticum* spores to heat treatment may have been caused by the action of bacteriocins. Information on interactions of *C. estertheticum* with companion cultures either in planktonic cultures or in biofilms is scarce. Regardless, the findings of the present study show that *C. estertheticum* can integrate into biofilms of other bacterial species and remain stable. This can aid the spores to disperse when biofilms go through their life cycle (O’Toole et al., 2000). The mechanisms of sensitization of spores by co-culture to heat warrants further exploration. Even so, the finding present a novel possibility of probiotic treatments by background microbiota or bacteriocins to limit the proliferation of free-living *C. estertheticum* or its establishment in biofilms in meat processing facilities.

Taken together, the findings of this study demonstrated that vegetative cells of *C. estertheticum* could withstand aerobic condition for up to 96 h and that spores were able to integrate into biofilms of other bacterial species. BioDestroy and UV-C LED can be effective control measures to eliminate spores of *C. estertheticum* either only surface attached or embedded in biofilms. The sensitization of spores of *C. esthertheticum* by oxidizing agents or companion cultures can be further explored as sequential treatment for BPS reduction.

## Data Availability

Sequencing data has been deposited under bioproject PRJNA1466567.
