# Supplementary material for: Fate of Clostridium estertheticum in biofilms and in response to conventional and novel sanitizers
Source: Front Microbiol. 2026 Jul 22;17:1840136. doi: 10.3389/fmicb.2026.1840136 (PMC13438422; doi:10.3389/fmicb.2026.1840136)
Supplement: Supplementary file 1 [file Table_1.docx]

**Figure S1.** Morphological variations of *Clostridium estertheticum* and *Pseudomonas poae* on a Columbia Blood Agar (CBA) plate: (A) After 3 weeks of anaerobic incubation at 10°C; (B) CBA plate exposed to flashlight illumination; (C) CBA plate was subjected to an additional week of aerobic incubation at 10°C.

**Figure S2.** Morphological variations of *Clostridium estertheticum*, *Leuconostoc gelidum*, and *Latilactobacillus sakei* on TSA and CBA plates: (A) After 72 h of aerobic incubation on a TSA plate at 25°C; (B) *C. estertheticum* and *Leu. gelidum* after 3 weeks of anaerobic incubation on a CBA plate at 10°C; (C) Mixed microbial consortia of *C. estertheticum*, *Leu. gelidum*, and *Lat. sakei* after 3 weeks of anaerobic incubation on a CBA plate at 10°C.
